# Response of dorsal horn neurons in mice to high‐frequency (kHz) biphasic stimulation is not sensitive to local temperature rise

**DOI:** 10.14814/phy2.70205

**Published:** 2025-02-06

**Authors:** Sergei Karnup, Stephanie Daugherty, Changfeng Tai, Naoki Yoshimura

**Affiliations:** ^1^ Department of Pharmacology & Chemical Biology University of Pittsburgh School of Medicine Pittsburgh Pennsylvania USA; ^2^ Department of Urology University of Pittsburgh School of Medicine Pittsburgh Pennsylvania USA; ^3^ Department of Bioengineering University of Pittsburgh Pittsburgh Pennsylvania USA

**Keywords:** kHz‐stimulation, lamina lucida neurons, spinal cord slices

## Abstract

Clinically accepted for treatment of chronic pain 10 kHz‐frequency electric spinal cord stimulation (10 kHz‐SCS) releases more power in tissue compared to conventional low‐frequency (<100 Hz) stimulation due to increased duty cycle. This is equivalent to the release of more heat in a surrounding tissue, which may change the functional state of affected neural elements. In the case of SCS, plausible candidates to be affected by thermal a component of kHz‐frequency electric field stimulation (kHz‐FS) are dorsal column axons and neurons of the superficial layers of the dorsal horn. In this study, we tested the hypothesis that joule heat produced by kHz‐FS modulates neuronal excitability. In slices of the mouse spinal cord, we monitored membrane potential and membrane input resistance in neurons of lamina II during exposure to kHz‐FS. Surprisingly, we found no correlation between temperature rise and changes of membrane parameters. Furthermore, the depolarizing effect of kHz‐FS was always immediate and remained persistent throughout stimulation, whereas rise of temperature was delayed for 1–2 s and reached its saturation level within the following few seconds. Thus, we concluded that the thermal component has an insignificant role in the mechanism of kHz‐FS action.

## 1 INTRODUCTION

Spinal cord stimulation (SCS) with trains of electric current pulses applied epidurally or subcutaneously is one of the therapeutic approaches to alleviate chronic or neuropathic pain (reviewed in Finnern et al., 2023; Fontaine, 2021; Gupta et al., 2022; Henson et al., 2021; Huang et al., 2019; Peeters & Raftopoulos, 2020; Sun et al., 2021). However, commonly used low frequencies (<100 Hz) SCS induce paresthesia (tingling, pricking, chilling, burning). In this respect, a novel 10‐kHz‐frequency therapy was shown to be superior to conventional SCS for the treatment of chronic back and leg pain (Kapural et al., 2015, 2016). The mechanism underlying low‐frequency SCS is hypothetically based on the gate‐control theory (Melzack & Wall, 1965), suggesting synchronous activation of Aβ axonal collaterals in the dorsal column, which activate a population of GABAergic inhibitory neurons in the dorsal horn. But it seems that paresthesia‐free subthreshold kHz‐SCS utilizes different mechanism(s) of action (MoA), which is presently unknown despite several hypotheses and mathematical modeling (Ahmed et al., 2018; Arle et al., 2016; Chakravarthy et al., 2018; Miller et al., 2016; Morales et al., 2019; Sagalajev et al., 2024; Vargas et al., 2023; Yi & Grill, 2020; Zhong et al., 2023). Recent experimental studies of this MoA suggest differential responsiveness of neurons to different frequencies. For example, differential modulation of excitatory and inhibitory neurons of the rodent dorsal horn by 10 kHz SCS accompanied by reduction of behavioral and neural hypersensitivity was demonstrated in vivo and ex‐vivo experiments in rodents (Lee et al., 2020, 2021, 2022; Wang et al., 2022, 2024). Studies at a neurochemical level have shown that in spared nerve injured rats prolonged administration of 10 kHz SCS alleviated chronic pain concomitantly with restoration of spinal glutamate concentration, frequency of mEPSPs, and significant suppression of ERK1, ERK2, JNK1, p38 in DRGs and dorsal horn (Liao, Tseng, Chia, & Lin, 2020; Liao, Tseng, Wu, & Lin, 2020). Computer modeling and recordings in spinal cord suggest that during paresthesia‐free 10 kHz SCS dorsal column axons are activated asynchronously unlike at conventional low‐frequency SCS which is supported by abolition of epidural evoked compound action potentials in pigs and rats (Sagalajev et al., 2024; Vargas et al., 2023). Among possible mechanisms of kHz‐frequency electric field stimulation (kHz‐FS), one can also consider joule heating by current passing through the tissue and cerebrospinal fluid. The computational and bath‐phantom modeling estimated a temperature rise up to 1.72°C at 3.5 mA peak 10 kHz current in the dorsal spinal cord (Zannou et al., 2019, 2021, 2022), and up to 1.38°C at 7 mA peak 10 kHz in the subthalamic nucleus (Khadka et al., 2020); temperature was shown to increase even higher at 500 Hz suggesting than the effect is related to power delivery and is not necessarily frequency specific (Zannou et al., 2023). Provided such heating effect of kHz‐FS modulates neural excitability and/or axonal conductivity or synaptic transmitter release, the kHz‐FS dose optimization might be a novel therapeutic pathway for treatment of chronic pain. In this paper we tested the hypothesis that temperature impact on the response of dorsal horn neurons to kHz‐FS may be essential for the SCS effect. In slices of the mouse spinal cord, we elicited responses of neurons in lamina II to external field stimulation at kHz frequencies concomitantly with temperature measurements near the site of recording.

## 2 MATERIALS AND METHODS

Male or female adult C57BL/6J mice were used for experiments. (2–6 months, 20–35 g). All procedures were approved by the Animal Care and Use Committee of the University of Pittsburgh. Mice were housed in the institutional animal facility and were fed with standard granulated food. Whole‐cell recordings were made from neurons of lamina II in slices of the spinal cord lumbar segments. To obtain slices, a mouse was anesthetized with isoflurane and decapitated. The vertebral column was excised and placed to a dish with ice‐cold sucrose‐based saline equilibrated with carbogen (95% O_2_ + 5% CO_2_): (in mM) 26 NaHCO_3_, 1 NaH_2_PO_4_, 3 KCl, 11 glucose, 234 sucrose, 10 MgSO_4_, 0.5 CaCl_2_. The vertebral column was pinned to a layer of silicon rubber on the bottom of the dish with the ventral side up. After excision of vertebrae's ventral portions, the spinal cord was gently extracted, a fragment containing L2‐L6 lumbar segments was dissected, sandwiched in a trough of the agar block, and 350 μm thick transverse slices were cut in the ice‐cold sucrose‐based saline on the vibratome Leica VT 1000S (Leica Biosystems, Buffalo Grove, IL). After cutting, slices were warmed at 32°–33°C for 0.5 h in the ACSF saturated with carbogen: (in mM) 117 NaCl, 26 NaHCO_3_, 3.6 KCl, 1.25 NaH_2_PO_4_, 2 MgSO_4_, 2 CaCl_2_, and 11 glucose; 285–290 mOsm, pH 7.4. Then slices were stored in this ACSF at room temperature (23°–24°C).

For whole cell recording, a single slice was placed in a chamber (~1 mL volume) and continuously perfused with ACSF at the rate ~3 mL/min. All recordings were performed at room temperature. The recording chamber was installed on an upright microscope (Olympus BX51W1, Life Science Solutions, Global) equipped for epifluorescence and near‐infrared differential interference contrast (DIC) optics. A CCD Hamamatsu C10600 ORCA‐R2 camera (Hamamatsu Photonics K.K., Japan) and MetaMorph software package (Molecular Devices, Sunnyvale, CA) were used for visualization of a recording electrode position. Patch pipettes were pulled from borosilicate glass capillaries with an inner filament (1.5 mm outer diameter; World Precision Instruments Inc., Sarasota, FL) on a pipette puller (P‐97; Sutter Instruments, Novato, CA) and were filled with a solution of the following composition (in mM): 114 K‐gluconate, 5 KCl, 0.5 CaCl_2_, 2 MgCl_2_, 5 HEPES, 5 EGTA. Osmolarity was adjusted to 270–275 mOsm, pH 7.3. The pipette resistance was 10–14 MΩ. Neurons and glial cells in lamina II were found blindly in voltage‐clamp mode at a depth 70–150 μm under the upper surface of a slice. Recordings were made in the current‐clamp mode with the MultiClamp 700B amplifier (Molecular Devices, Sunnyvale, CA) and data acquisition software package Signal5 (Cambridge Electronic Design Limited, Cambridge, UK). There was no correction for the liquid junction potential (~10 mV).

**FIGURE 1:**
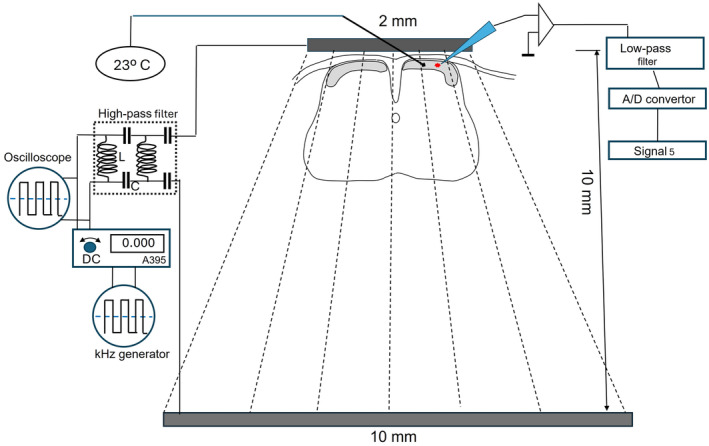
Experimental setup for concomitant whole‐cell recording from lamina lucida neurons and measurement of temperature in a slice of spinal cord. Parallel stimulating electrodes on the bottom of the chamber were 10 mm apart, and a slice was placed immediately near the short electrode. Optical thermistor (100 μm diameter) was positioned on a surface of a slice next to a recording site. Trains of kHz‐frequency biphasic pulses were delivered through a high‐pass filter to eliminate possible DC contamination. Recorded in current‐clump membrane potentials were low‐passed to eliminate kHz frequencies induced by stimulation.

Responses of cells to kHz field stimulation were obtained in the submerged mode, where most of the current was shunted by surrounding ACSF. Therefore, a portion of current passing through a slice or through a cell could not be precisely determined. Trains of kHz‐frequency rectangle balanced biphasic pulses were applied between two flattened parallel stainless‐steel bars glued to the bottom of the chamber. Both bars were 1 mm wide and 300 μm thick with 10 mm distance between them. However, they had different lengths: 2 and 10 mm to create an anisotropic electric field. A slice was positioned with its dorsal side in the vicinity of the short bar at a distance 100–500 μm where the current density was the highest (Figure 1). Importantly, in our experiments ACSF flew in the direction from the short stimulating electrode to a slice.

Therefore, while passing the short bar, the ACSF flow was heated and then delivered to a site of recording. This differs from situation in vivo where heat dissipates passively in surrounding tissue. However, this approach allows us to assess the effect of the thermal component of kHz‐FS. To control temperature accompanying passing current, we used optical thermistor OTG‐F insensitive to electric fields (Opsens Solutions, Canada). Diameter of the thermistor was 100 μm, so it could be positioned at the surface of a slice near a recorded cell (~100 μm distance on the line parallel to the bar). A kHz‐frequency train of balanced biphasic square pulses was generated by the arbitrary function generator AFG 2105 (GwINSTEK). Pulse width “t” (a half of the biphasic wave width “2 t”) was not adjustable, so that *t* = 1/2ω, where ω is stimulation frequency. To convert voltage pulses into current pulses, a stimulus isolator A395 (WPI) was used. To eliminate a possible DC component of stimulating currents, we inserted a high‐pass filter in the stimulating circuit (Franke et al., 2014). To eliminate high‐frequency stimulating waveform from intracellular recordings and avoid aliasing artifacts, the 900 Tunable Active Filter (Frequency Devices, Inc.) with sharp low‐pass characteristics was inserted in the data acquisition circuit. Its cutoff frequency was set to values twice as low as a stimulating frequency; this completely prevented kHz‐FS contamination of the recorded signal, but still allowed for registration of spike occurrence (in our case, detailed spike shape is not important). Sampling frequency was 10 kHz. Some cells were tested with only 1 kHz‐frequency, but most cells were tested with two or more frequencies in the range between 2 and 10 kHz to compare efficacy of different frequencies.

We recorded in whole‐cell current clump configuration responses of unidentified neurons to externally applied kHz‐FS. To monitor input resistance (R_in_) a 500 ms pulse of negative current was injected via the microelectrode every 20 s. R_in_ was calculated by dividing the amplitude of the maximal voltage deflection within the first 200 ms to the magnitude of injected current (usually 50 or 100 pA). To monitor rheobase (Rh), a minimal pulse of positive current eliciting a spike was regularly applied. This minimal injected current was accepted as the value of rheobase.

In mouse spinal cord slices we blindly patched and recorded 18 neurons in lamina II of the dorsal horn. Three basic stimulation frequencies were 2, 5, and 10 kHz. However, sometimes other frequencies within the 2–10 kHz range were used. Stimulation amplitudes were in the range of 2–10 mA because 2 mA were either just threshold or subthreshold at all frequencies, and 10 mA led in most cases to a strong irreversible depolarization. Field stimulation was performed with trains of kHz‐frequency rectangle balanced biphasic pulses. Duration of trains was typically 3–5 min. However, in some cases stimulation was canceled earlier when a cell was overexcited, showing rapidly increasing depolarization which often became irreversible after reaching V_m_ > −20 to 30 mV. We considered depolarization irreversible if V_m_ > −20 mV for more than 10 min (duration of a recorded sweep). Three strongly depolarized neurons monitored for 20 min did not recover; instead, they completely lost V_m_. Every frequency/amplitude composition was tested prior, during and after exposure to kHz‐FS. In some cases, the same composition was repeated once or twice to reveal adaptation or variability of responses.

## 3 RESULTS

**FIGURE 2:**
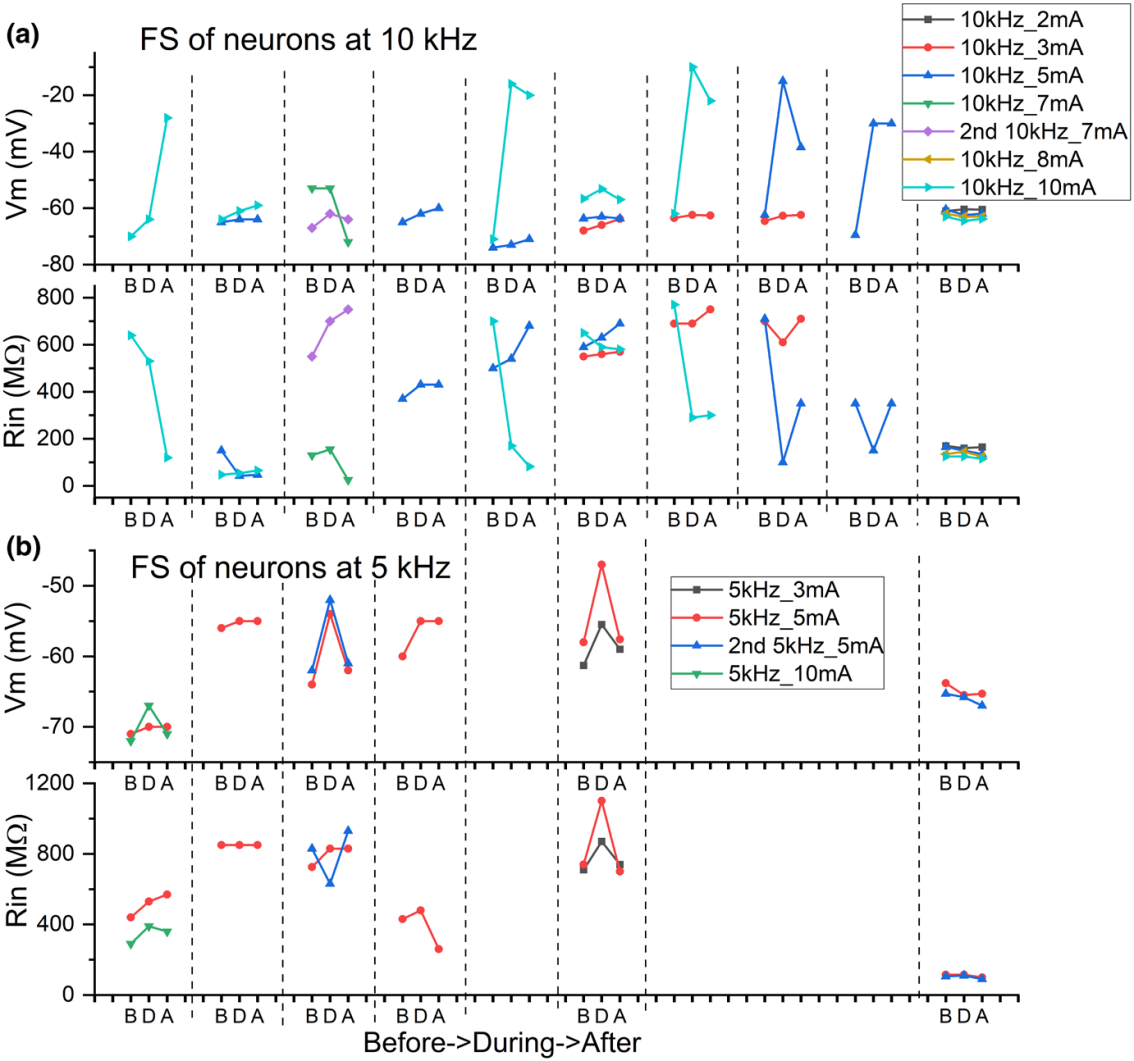
Illustration of response variability among a group of neurons. Each column of 3‐dot plots between vertical dotted lines extending through a and b reflects changes of V_m_ and R_in_ in the same neuron before, during, and after kHz‐FS. (a) Stimulation at 10 kHz with different currents amplitudes. (b) Stimulation at 5 kHz with different currents. FS, field stimulation; LL, lamina lucida.

**FIGURE 3:**
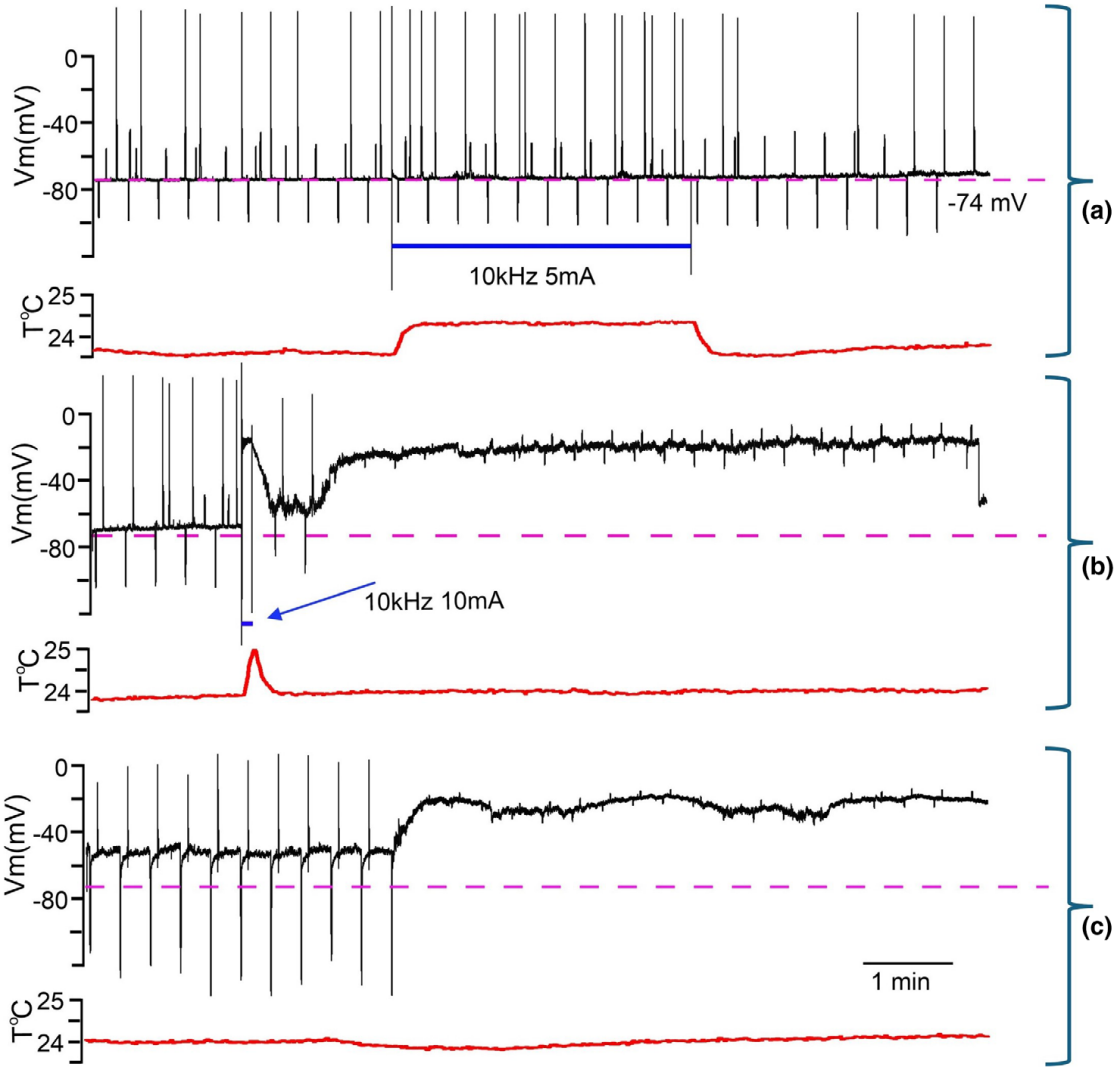
Three consecutive sweeps, each 10 min long, show responses of a neurons to 10 kHz with 5 and 10 mA currents. Black traces designate V_m_, red traces designate temperature near a recorded cell. Dotted magenta line indicates the initial V_m_, and solid blue line shows periods of kHz‐FS. Solid blue line indicates duration of kHz‐FS. Throughout the recording –50 pA and +50 pA pulses were injected every 20 s to monitor input resistance (R_in_). Additional positive pulses of current were injected to monitor rheobase (Rh). (a) kHz‐FS with 10 kHz/5 mA eliciting negligible response. (b) kHz‐FS with 10 kHz/10 mA immediately resulting in an abrupt depolarizing shift following by a long period of strong depolarization and low of R_in_. (c) Aftereffect of stimulation showing a temporary (and partial) recovery of V_m_ with restoration of spiking and with a large increase of R_in_ followed by the second switch to suprathreshold depolarization with loss of R_in_. Note that in B temperature rise did not reach its maximum and was brief, whereas physiological response of the cell was stronger than in A.

**FIGURE 4:**
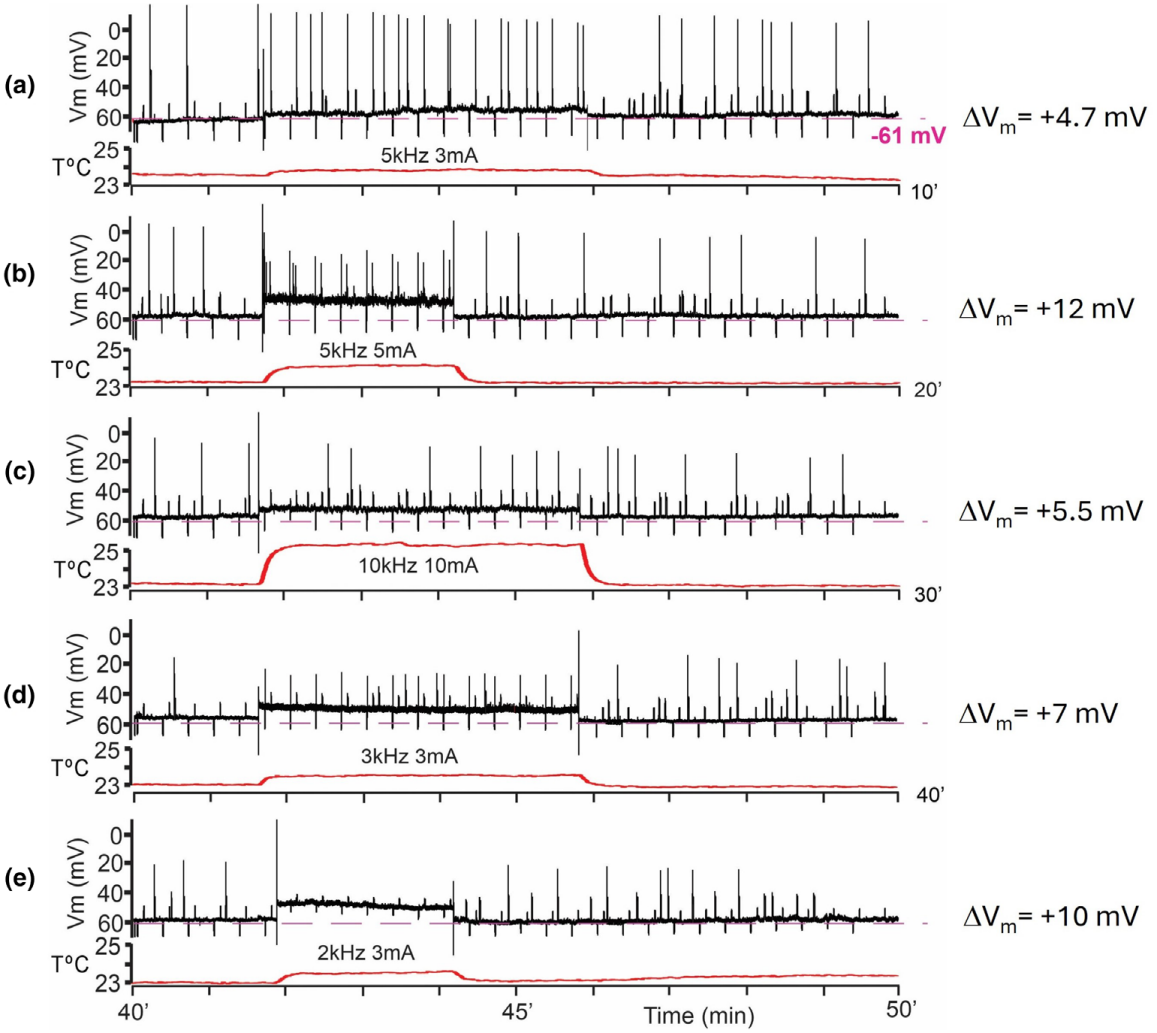
Five consecutive sweeps of a neuron stimulated with different frequency/amplitude combinations. Black traces designate membrane potentials (V_m_), and red traces stand for temperature measurements. Solid blue line indicates duration of kHz‐FS. Magnitudes of depolarizing shifts of the membrane potential are shown on the right of each plot. Note that responses to all frequency/amplitude combinations were fully reversible. Dotted magenta line designated the initial value of V_m_. Throughout the recording −20 pA and +20 pA pulses were injected every 20 s to monitor input resistance (R_in_). Additional positive pulses of current were injected to monitor rheobase (Rh).

**FIGURE 5:**
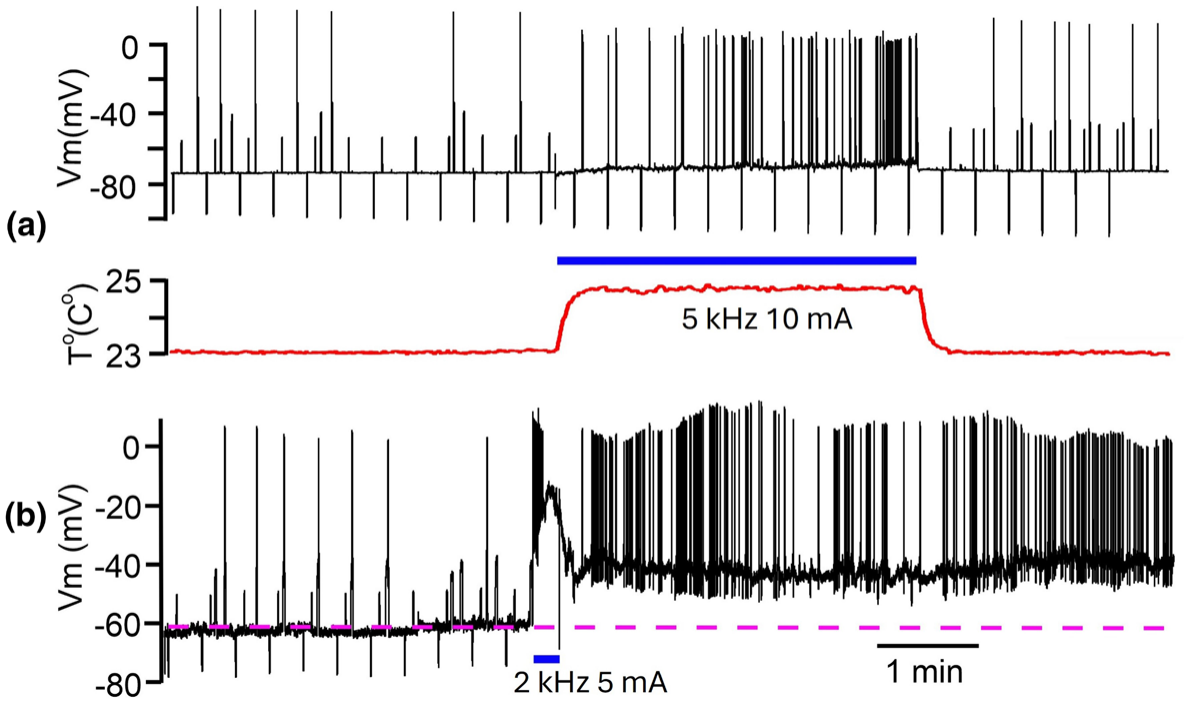
Examples of responses to kHz‐FS. (a) Gradual depolarization with increasing spontaneous firing at 5 kHz 10 mA. (b) At 2 kHz, 5 mV elicited an immediate vigorous depolarization which ended after FS termination, but the V_m_ stayed depolarized with active spontaneous firing.

Within the recorded population, neurons demonstrated variability of response expression to the same or similar composition of stimulus' parameters (Figure 2). Moreover, responses of a neuron could differ from the first to the second presentation of the same frequency/amplitude combination (3rd column from the left in Figure 2). However, during stimulation in almost all cases we saw a depolarizing shift of V_m_. After termination of kHz‐FS V_m_ could return to pre‐stimulation value or could stay at a more depolarized level. Strong stimulation often led to irreversible depolarization. Due to different sensitivity and resilience of neurons to stimulating currents, it was not possible to predict what amplitude of stimulation at a given frequency would be suprathreshold or would be damaging to a cell or would cause irreversible changes of membrane parameters. For example, when those cells which were stimulated by 10 kHz were divided into 3 groups according to applied current amplitudes, they showed a substantial variability of responses in each group. At 10 kHz with 3 mA, 1 of 8 cells showed ΔV_m_ = −8 mV, whereas other 7 cells did not respond to 2–3 mA currents. At 10 kHz and 5–6 mA, 3 of 13 neurons responded with a reversible depolarizing V_m_ shift, 4 neurons showed a small (−1 to −2 mV) reversible hyperpolarizing shift, and 6 cells did not respond (Figure 3a). Of 9 neurons tested with 10 kHz/10 mA three cells responded with a subtle depolarization (Figure 4c), 6 cells showed strong or moderate depolarizing shift of V_m_; 4 cells did not recover from strong depolarization and kept V_m_ = −20 to −30 mV to the end of recordings (for >5 min) (Figure 3b,c). Stimulation at 5 kHz with 2–3 mA was always suprathreshold in terms of V_m_ shifts either up to spike generations or further up to spike depolarizing block (Figure 4a); but this frequency/amplitude combination never led to an irreversible depolarization. Stronger stimulation with 5 kHz/5 mA caused 10–12 mV depolarization in 4 of 6 neurons (Figure 4b), a subtle −2 mV hyperpolarization in one neuron and did not induce a response in one neuron; in none of the cases an irreversible depolarization occurred. Only one cell was recorded during stimulation with 5 kHz/10 mA which caused gradually increasing spontaneous firing (Figure 5a). Other cells stimulated with 5 kHz/10 mA immediately lost their physiologically relevant V_m_ and never recovered (their recordings were discarded). Stimulation with 2–3 kHz had on average the lowest thresholds, and even at moderate current amplitude of 5 mA resulted in an immediate strong depolarizing V_m_ shift and decrease of R_in_ (Figure 5b). At high stimulation intensities of 9–10 mA 2–3 kHz always caused up to 10 min depolarization above −20 mV and the following complete loss of V_m_ to 0 mV. That is why we collected only a few records at the lowest kHz‐frequency. In an instance when 2 kHz‐FS/5 mA was rapidly terminated preventing lasting depolarizing block and the following Ca^2+^ intoxication, a neuron could stay at suprathreshold V_m_ spontaneously firing for half an hour but did not recover to its initial state despite partial R_in_ restoration. It seems that from the selected range the lowest kHz frequencies result in the harshest and often damaging (i.e., unrecoverable V_m_ = 0 mV for minutes) effect. At the same time, 10 kHz seems to be the gentlest frequency which allows easier tuning of current amplitude to obtain sensible response, but even at high (up to10 mA) current amplitudes, it did not cause a complete loss of membrane potential to 0 mV; instead, V_m_ remained at the level −20 to −30 mV which inactivates fast sodium spikes but has a potential to recover.

**FIGURE 6:**
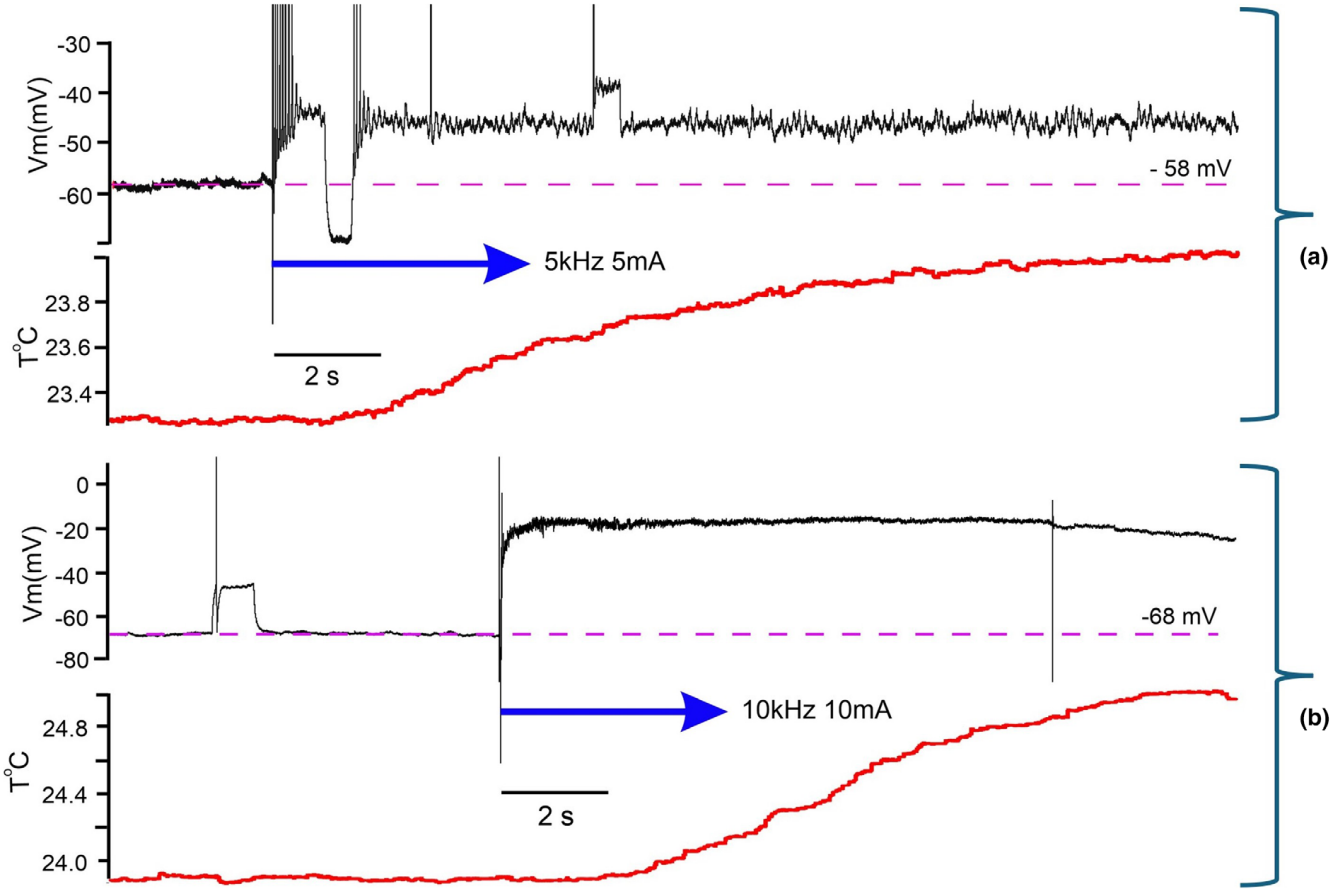
Initial fragments of responses to kHz‐FS showing an instant reaction of a neuron to kHz‐FS and a gradual increase of temperature at the site of recording. The temperature rise is delayed for 1–2 s, and the plateau of V_m_ is not deformed while temperature increases. (a) is taken from Figure 4b is taken from Figure 5b. Spikes are truncated. In (b) kHz‐FS starts with the blue arrow and lasts till the vertical line on the V_m_ trace representing the “turn off” artifactual pulse.

The first and the most convincing feature of the neuronal response suggesting its independence of joule heat produced by passing current is the almost instant rise of V_m_ to a magnitude staying persistent throughout kHz‐FS exposure, whereas temperature rise is delayed for ~2 s and then exponentially rises during 20–30 s (Figure 6). Furthermore, since in this case stimulation was interrupted before the full development of its effect (see Figure 3b), depolarization started weakening immediately after cessation of kHz‐FS, whereas temperature was still rising (Figure 6b). Obviously, an increasing distance from the short bar to a recorded neuron would increase the delay of heat delivery, but at the same time it would weaken the electric field effect. In this work we were interested in proof of the concept, so we did not make detailed study of response distance‐dependency.

**FIGURE 7:**
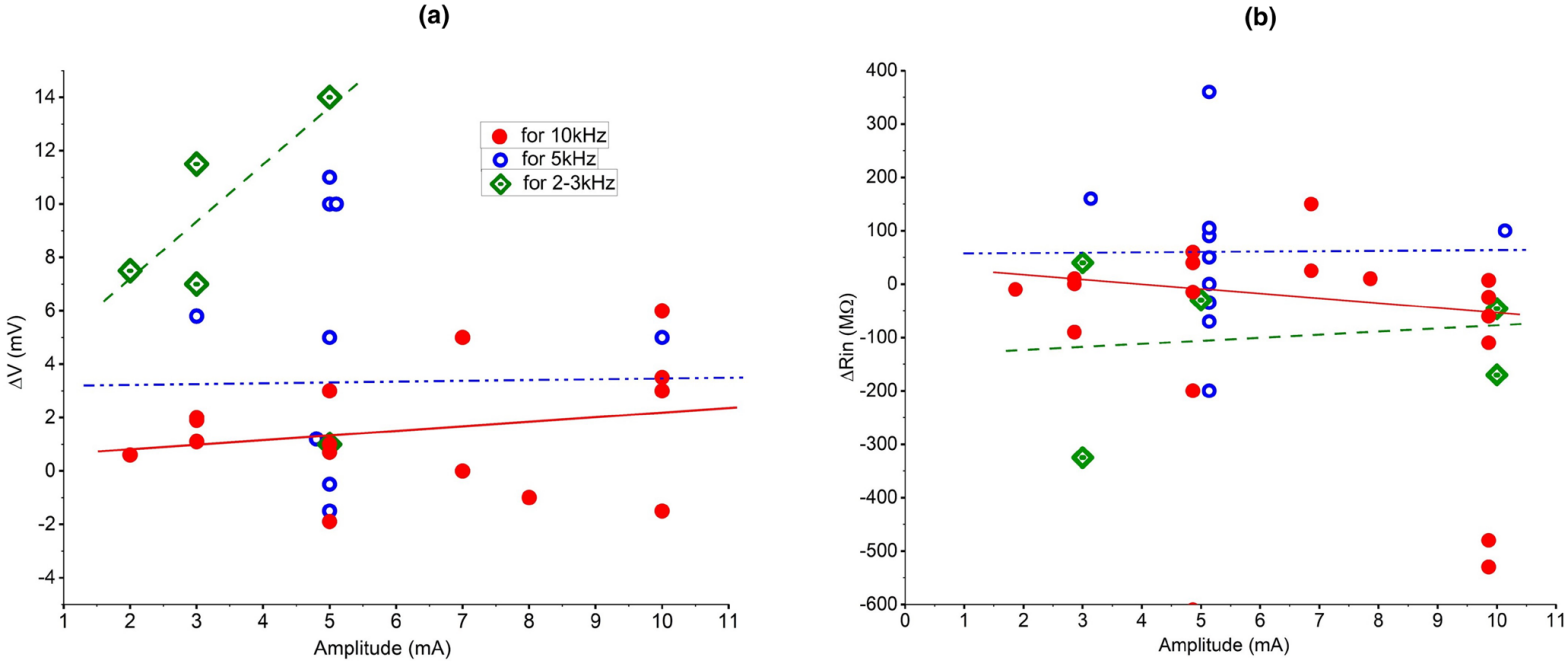
Changes of V_m_ (a) and R_in_ (b) with increasing current. At 10 kHz, even with the strongest stimulating current the response magnitudes are modest (red solid circles), and their increase is more gradual with the increase of current as compared to 5 and 2 kHz. At 5 kHz depolarization can be higher when current amplitude is half of the maximum (blue hollow circles). At 2 kHz pronounced reversible depolarizing V_m_ shifts occur even to the lowest tested currents (green hollow diamonds with the central dot); cases with response‐related irreversible depolarization are not shown. Each symbol stands for an individual stimulation episode (not for an individual neuron). Regression lines have the same color as symbols: Red solid line is for 10 kHz, blue dash‐dotted line is for 5 kHz, and green dashed line is for 2–3 kHz.

In our experiments, 10 kHz‐FS induced either sub‐ or suprathreshold depolarization of cells, but never caused irreversible shifts of membrane potentials. The 5 kHz‐FS and 2–3 kHz‐FS with moderate or strong currents could result in a permanent depolarization shift above the spike‐inactivating V_m_ with higher probability. The scattered plot in Figure 7a shows an obvious but not steep V_m_ change with an increasing stimulating current from the lowest to the highest amplitude at 10 kHz. At 5 kHz and half‐maximal currents, ΔV_m_ can reach a greater magnitude than at 10 kHz with even the maximal current. Finally, at 2 kHz a steep enhancement of the response occurs while stimulating current rises with small increments (responses ended with permanent depolarization to >−20 mV are not shown). In other words, at 2 kHz, a sudden, strong, and probably massive depolarization may result in an immediate impairment of the most fragile neuronal elements just after turning the kHz‐FS on. Thus, the impact of current amplitude in an average physiological effect diminishes with increasing kHz frequency.

**FIGURE 8:**
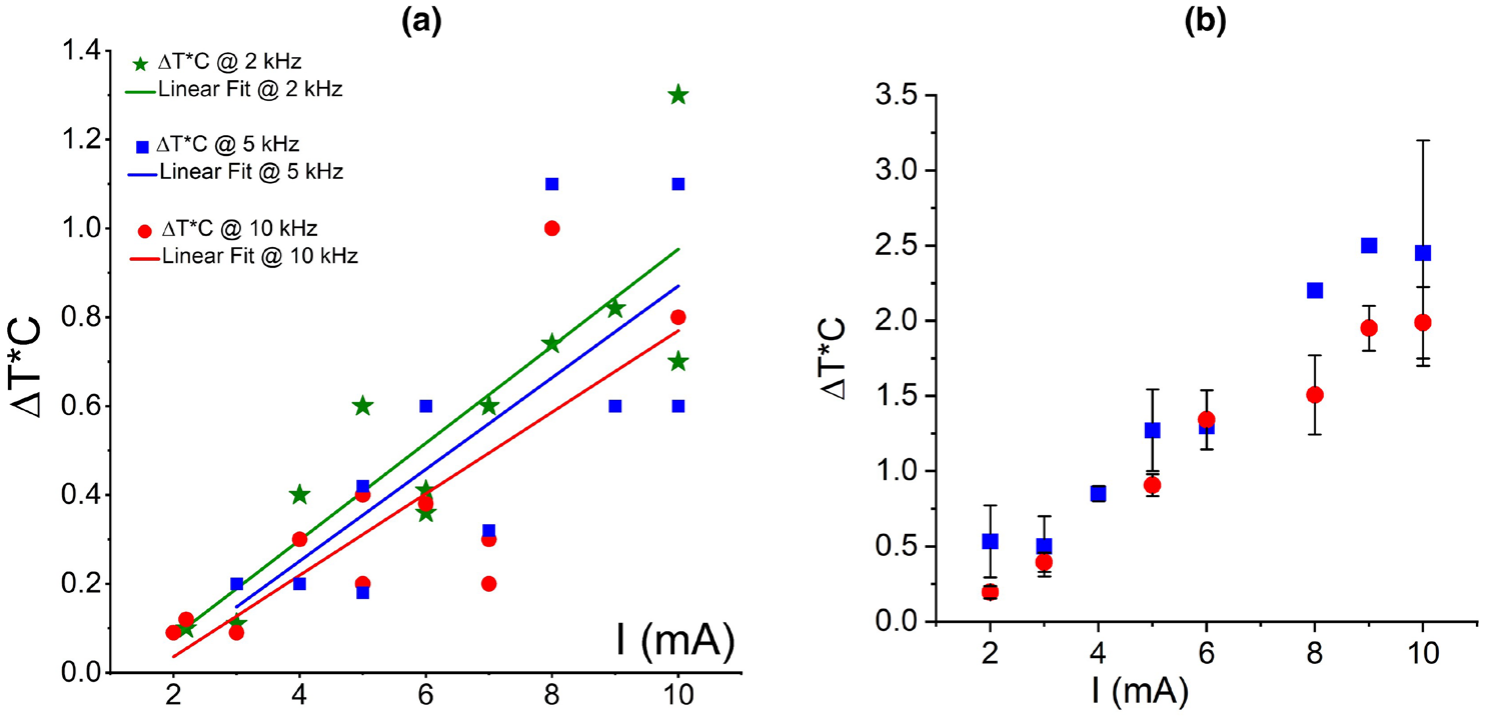
Scattered plots of temperature change vs. current amplitude during stimulation. (a) Model experiment with ΔT°C measurements at varying distances of 1.0 ± 0.5 mm from the short bar. (b) Averaged temperature changes collected from the whole‐cell experimental data. Red circles – 10 kHz, blue squares – 5 kHz, green stars (in a only) – 2 kHz.

When comparing amount of heat delivered by ACSF flow to the slice one can notice that rise of temperature is dependent on amplitude of stimulating current but not so much on frequency (e.g., Figure 4a,b; Figure 3a and imaginary extrapolated sharp rise of T°C in Figure 3b). Indeed, temperature rise following incremental increase of kHz‐FS current magnitude shows linear correlation for both 10 and 5 kHz (Figure 8b). The linear regression approximation indicated that around 7–8 mA temperature had risen to ~ +1.5°C to +1.7°C, which is comparable with physiologically relevant estimates in models (Khadka et al., 2020; Zannou et al., 2021). Data points corresponding to 5 kHz had a somewhat steeper alignment than points of 10 kHz, which suggests a weak reverse correlation of released heat and frequency of stimulation. To confirm this observation, we performed a model experiment without whole‐cell recordings (Figure 8a). We measured temperature changes in the ACSF during 2, 5, and 10 kHz stimulation with 2–10 mA currents while randomly placing the thermistor within 1.0 ± 0.5 mm distance from the short bar. These distances were somewhat larger than those used in whole‐cell experiments; therefore, temperature changes were of lower magnitudes due to dilution of a heated ACSF portion by surrounding non‐heated solution. In both cases, temperature rise showed linear correlation with current amplitude. It also showed a slight reverse correlation with frequency of stimulation which is indicated by slopes of linear regression in Figure 8b. Thus, joule heat produced by trains of balanced kHz‐frequency biphasic pulses poorly depends on frequency (in the range 2–10 kHz) but strongly correlates with amplitude of current. The discrepancy between a weak correlation of ΔV_m_ with current amplitude on one hand, and a linear rise of ΔT°C with increasing current at 10 kHz on the other hand, implies that MoA of kHz‐FS is not a mere effect of electromagnetic field energy transformation into heat‐generating movement of charged molecules/ions which takes place when current passes a resistive medium (i.e., ACSF).

**FIGURE 9:**
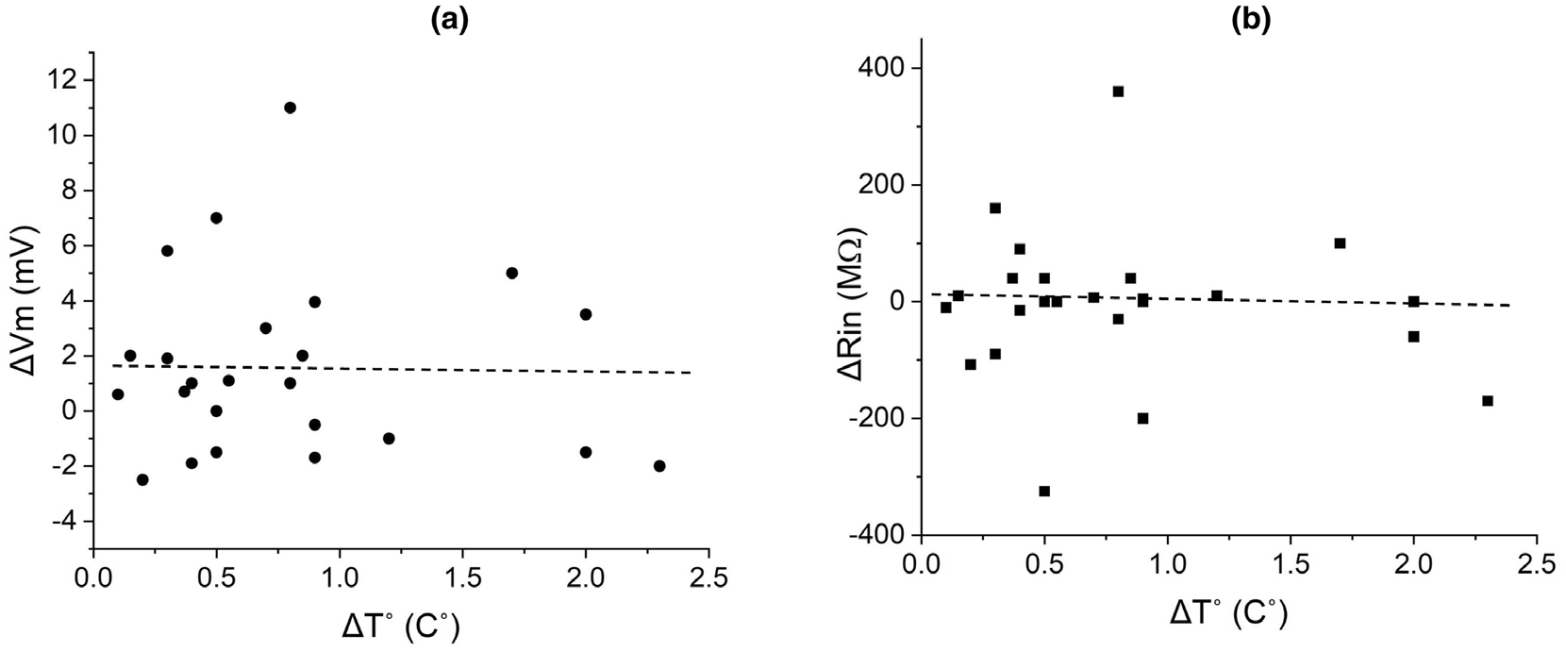
Distributions of ΔV_m_ (a) and ΔR_in_ (b) show a lack of correlation with ΔT°C during kHz‐FS. Each data point stands for an individual response (not an individual neuron).

Finally, when we considered only the rise of temperature near electrodes regardless of other parameter of stimulation, the scattered diagram of ΔV_m_ and ΔR_in_ against ΔT°C showed no correlation between membrane parameters and temperature changes (Figure 9).

## 4 DISCUSSION

The mechanisms of kHz (>1 kHz) spinal cord stimulation are still unknown and remain under investigation (Kapural et al., 2016; Mendell, 2014; Zhang et al., 2014). Distinct clinical traits of kHz‐SCS imply that its mechanisms of action (MoA) differ from that in conventional low‐frequency stimulation (Crosby et al., 2017; Lempka et al., 2015; Maeda et al., 2009; Song et al., 2014). In kHz‐SCS, the highly resistive epidural space plays a central role in temperature increases both at lead and at the spinal cord (Zannou et al., 2019). The layer of the cerebrospinal fluid (CSF) between the dura mater and the dorsal surface of the spinal cord (subarachnoid space) is a good conductor for spreading heat. MRI data obtained in humans show that within the T6‐L1 range the thickness of CSF layer increases almost linearly by 0.5 mm per vertebra from 3.0 mm at T12 to 5.8 mm at T6 (Holsheimer et al., 1994) allowing easy diffusion and heat transfer. Thus, superficial layers of the dorsal horn may be affected by the thermal component of kHz‐SCS by passive spread of heat through CSF and tissues. In our experiments it was the ACSF flow that delivered heated solution to a slice, but in principle the effect is the same as in vivo. Technical limitations for in vivo temperature measurements in tissue of the spinal cord during application of epidural kHz‐FS prevent direct comparison between the pure electric field effect and the pure thermal effect on neuronal excitability. Until now, all assessments of heat spread were done by mathematical modeling, and an impact of thermal component was considered only hypothetically (Elwassif et al., 2012; Khadka et al., 2020; Zannou et al., 2019, 2021). In animal models significant brain excitability was observed with short‐term increases of >2°C (Harris et al., 1962; Kim & Connors, 2012; Matsumi et al., 1994). However, a sustained 1–2°C rise in brain temperature after injury is potentially hazardous (Childs, 2008; Childs et al., 2008; Dietrich, 1992; Morikawa et al., 1992). It is also not clear to whether a transient but long‐term kHz‐FS exposure subthreshold for neuronal excitation can produce enough heat to affect local physiologically important functions such as ion gating and transmitter release/clearance or to affect specific molecular pathways. Slow temperature‐dependent homeostatic changes may provide a plausible explanation for the delayed relief by kHz‐SCS (Al‐Kaisy et al., 2015; Arcioni et al., 2016; Thomson et al., 2018).

It seems that the reason why 10 kHz has been empirically chosen as the optimal frequency for kHz‐SCS is the fact that 10 kHz‐FS provides a more gradual enhancement of physiological response with an increasing current. This allows for a safer adjustment of stimulation strength and a lesser probability of an overdose. On the contrary, kHz‐SCS with lower frequencies can much easier switch the neuronal status to inactivation or cause irreversible impairment even at moderate current amplitudes. Therefore, in case of kHz‐FS, an applied frequency is the decisive parameter of stimulation. Since the capacitive‐resistive properties of the lipid bilayer membrane serve as a low‐pass filter, they reduce the power of high‐frequency components of the stimulus up to total elimination (Hutcheon & Yarom, 2000). One can assume that the 2–10 kHz window is close to the edge of the neuronal membrane low‐pass filtering bandwidth, so that frequencies of hundreds of kHz should be ineffective whereas frequencies of a few tens of kHz still may result in a weaker physiological effect. Another possible MoA of kHz‐FS may be an interaction of electric field with certain molecules or molecular groups in the cellular membrane modulating permeability of voltage‐independent ion channels (e.g., leak channels). In this case, the classical representation of the membrane as a set of capacitor/resistor couples embedded to the lipid bilayer should be supplemented with a set of sensors selectively affected by 1–10 kHz bandwidth and capable of gating leak channels.

One should always be cautious about possible artifacts induced by instruments. For example, DC component of kHz‐FS generated by even slightly disbalanced stimulus isolator can itself cause polarization of cells. To avoid DC contamination, we inserted a high‐pass filter (Franke et al., 2014) between A395 and stimulating electrodes. Another cause of an instrument‐related artifact was discovered by Prescott's group (Lesperance et al., 2018). They observed kHz‐FS‐induced hyperpolarization of −20 to −40 mV in patched neurons while recording in current‐clamp mode with Axopatch 200B amplifier. The hyperpolarization had an exponential decay lasting ~100 ms and an elaborate profile of ΔV_m_ against stimulating current. However, if the Axoclamp 2B was used, the hyperpolarizing shift of V_m_ was absent, suggesting that its occurrence is related to a type of amplifier: patch‐clamp type versus voltage‐follower type. We recorded V_m_ with MultiClamp 700B. In our recordings, hyperpolarizing responses did not exceed −2 mV and occurred less frequently than depolarizing ones at the same current amplitudes (see Figure 7a). Thus, we conclude that there was no systematic skewing of data by our amplifier. A complete exclusion of instrument‐induced data distortion can be done in a special series of experiments. For example, by using cell culture and voltage‐sensitive dyes (Lesperance et al., 2018).

Although the mechanism(s) of delayed effects of kHz‐FS cannot be assessed in a slice like it is possible to do in vivo, the ex vitro approach allows for separate assessment of potentially relevant parameters of stimulation as well as of correlations between them. In this publication we tested a possible role of kHz‐FS‐related temperature rise in modulating general characteristics of neuronal membranes like V_m_ and R_in_. Surprisingly, the degree of neuronal depolarization and changes in input resistance during kHz‐FS was totally independent of heat which was generated in the vicinity of the stimulating electrode. Thus, based on the obtained data, we made two conclusions. First, during relatively short stimulation (<10 min), spinal neurons are not sensitive to a byproduct of the kHz frequency electric field, such as joule heat. Therefore, the thermal component of kHz‐FS can be excluded from consideration in a search for an actual mechanism of kHz‐FS action. Second, sensitivity of neurons to amplitude of stimulating current depends on kHz‐frequency used for stimulation. Stimulus strength seems to be much easier to tune for the optimal effect at 10 kHz compared to lower frequencies of kHz range. On the other hand, higher than 10 kHz frequencies may require stronger currents and may be less effective.

## AUTHOR CONTRIBUTIONS

S.K. designed the study, performed experiments and data analysis, wrote the original draft; C.T. and N.Y. discussed the manuscript; S.D. helped with animals and general organization of work.

## CONFLICT OF INTEREST STATEMENT

The authors declare no conflict of interest.

## ETHICS STATEMENT

All animal experimental procedures performed in this study were approved by the Animal Care and Use Committee at the University of Pittsburgh School of Medicine, Pittsburgh, PA.

## PUBLICATION DISCLAIMER STATEMENT

The findings and conclusions in this report are those of the authors and do not necessarily represent the official position of the NIH.

## Data Availability

The data that support the findings of this study are available from the corresponding author upon a reasonable request.
